# Forecasting climate-associated non-tuberculous mycobacteria (NTM) infections in the UK using international surveillance data and machine learning

**DOI:** 10.1371/journal.pgph.0003262

**Published:** 2024-08-19

**Authors:** Amy Marie Campbell, Katy Willis, Edward Parsons

**Affiliations:** Global Assessment and Emerging Hazards Division, UK Health Security Agency, London, United Kingdom; University of Oslo Faculty of Medicine: Universitetet i Oslo Det medisinske fakultet, NORWAY

## Abstract

Nontuberculous mycobacteria (NTM) cause skin and lung infections, have high mortality rates, and are resistant to a range of antibiotics and water treatment methods. As NTM reside in environmental reservoirs, they are sensitive to environmental conditions. The suitability of their environmental reservoirs can increase as a result of climate change, subsequently increasing environmental exposure and infection rates. NTM infections are not generally notifiable, including in the UK, but sustained increases have been observed in regions that report NTM infection rates. To assess the burden of NTM infections in the UK under projected climate change, we examined the relationship between climate variables and available NTM surveillance data internationally. Statistically significant increases were found in regions where NTM infections are notifiable, which were positively associated with increased precipitation and temperatures. A random forest regressor was trained using supervised learning from international NTM surveillance data and linked climate variables. The random forest model was applied to UK climate projections, projecting a 6.2% increase in NTM infection rates over the next 10 years, with notable regional variation. Our random forest model predicts that the forecasted impacts of climate change in the UK, including increasing temperatures and frequency of heavy rainfall, will lead to increases in NTM infection rates. Robust surveillance in the future is necessary to increase data available to train models, increasing our predictive power in forecasting climate-associated NTM trends. Our results highlight a novel aspect of how climate change will impact health outcomes in the UK.

## Introduction

Nontuberculous mycobacteria (NTM) are a group of bacteria that can cause lung and skin infections in humans when transmitted via ingestion of contaminated water, aerosols, and soil [1, 2]. NTM are found in the environment, residing in natural water bodies, drinking sources and soil [3]. There are at least 180 NTM species, of which 25 species can cause illness in humans [4], with pulmonary NTM (PNTM) infections being the most common form of illness [5]. Such infections carry a high mortality and disease burden, requiring an 18-month course of antibiotics [6] which still leads to a 50% reinfection rate [7]. Additionally, NTM infections have a poor prognosis, with an average 5-year mortality rate of 27%, increasing up to 48% in patients with comorbidities [6].

It has previously been difficult to assess the specific exposure and risk of NTM, due to the significant lag between exposures and diseases presentations [8], but there is a growing need to solve the research gaps of this commonly-neglected but dangerous pathogen. Particularly of note are the biological traits that make NTM distinct from any other microbe, such as their unprecedented capacity for adaptation, allowing NTM to colonise a large diversity of environments [3] and facilitating the emergence of such an opportunistic pathogen. Specifically, NTMs are recognised by WHO as being a pathogen that is of relevance for drinking-water supply management [9], particularly as one of the most resistant bacteria [9], referring to their resistance to chlorine water treatment and many antibiotics. In the last decade, NTM infections have been attracting attention in medical communities, with NTM infections starting to outweigh the number of tuberculosis infections in developed countries [3]. While previously associated with immunocompromised patients [3], PNTM infections are now increasing in immune-component people worldwide [6]. The incidence of NTM is increasing globally for both infection and disease [10], with a systematic review reporting 38 of 47 studies observed an increasing trend in NTM infection or disease, that is not explained by increases in surveillance [10]. These studies spanned 18 countries, covering a range of geographical locations in Asia, Europe, North and South America[10], highlighting the global extent of the burden.

*Mycobacterium* species are capable of growing at a wide range of temperatures, dependent on species. Optimal growth rates of laboratory cultures were found between 28°C and 37°C, with some disease-causing species able to survive in the environment at temperatures up to 55°C [11]. Impacts of urbanisation can drive increases in shallow groundwater temperatures by 3°C compared to rural areas, providing a more supportive environment for NTM survival and growth [12]. Increased levels of NTM in treated municipal water supplies have been observed during warmer months in Mexico City [13] and the Netherlands [14]. A laboratory study on *M*. *avium*, the most prevalent infection-causing NTM, in biofilms in potable water systems concluded that survival was more sensitive to temperature than nutrient availability [15].

Additionally, precipitation is a complex driver of NTM in the environment, both increasing or decreasing NTM presence depending on coincident environmental conditions. Heavy precipitation, and associated surface runoff, turbidity and nutrient flows, can increase the abundance of NTM in surface waters [12]. A study of NTM notification rates and local climate data in Queensland, Australia found NTM incidence positively correlates with peaks in rainfall after a 9 month lag for slow-growing *Mycobacterium* species, and a 4–5 month lag for fast-growing species [8]. Reduced precipitation, and subsequent reduced water table levels, drives a decreased flow of colder water from upstream, increasing surface water temperatures and favouring NTM growth [12]. Conversely, reduced river volume in estuary environments would result in salt-water intrusions that decrease environmental NTM presence, due to salinity stress [16]. In the US, spatial cluster analysis found regions with high potential evapotranspiration levels and high percentage surface water coverage have an increased risk of PNTM infection [17].

There have been multiple calls to expand and improve laboratory research, epidemiological studies and global data collection of NTM disease in order to better inform decision-makers (e.g. [3, 10, 18]). Mandatory reporting of NTM disease is uncommon, and NTM infection is not notifiable in the UK, with a study last inferring NTM infection rates at 7.6 infections per 100,000 people in the UK in 2012, from clinical laboratory isolates [19]. Regions that have made NTM infections notifiable include Finland; Queensland, Australia; and 14 of the US states that require some form of NTM infection reporting (S1 Fig). Given that climate change scenarios predict an increase in average temperature and potential changes in precipitation patterns [20], it is likely that NTM incidence will change. To understand potential changes in NTM infection rates in the UK over the next ten years, we examined the relationship between global climate variables and available NTM incidence. We use these relationships to forecast future NTM infection rates in the UK under climate change scenarios.

## Methodology

We obtained NTM infection data from the following regions and time-periods where the disease is notifiable: Finland, 1995–2022 [21]; and Mississippi, US, 2005–2020 [22], and from where isolates are routinely collected: Scotland, 2011–2019 [23]. Mississippi and Finland were selected due to the accessibility of surveillance data of NTM infections, rather than their completeness as comparator countries for the UK, however in combination these countries offer a sufficient coverage of the UK’s temperature and precipitation ranges (S2 Fig).

Monthly NTM infection data from Mississippi was aggregated to a yearly resolution to match the Finnish and Scottish data resolution. Raw numbers of infections were normalised to a more comparable metric of infection rate per 100,000 people, based on population data for each region [24]. We calculated Pearson’s correlation coefficient (R^2^) between the NTM incidence rates and time for each region, to assess whether NTM infections were significantly increasing over time.

We obtained historic temperature and precipitation data for the time-periods overlapping the available NTM infection data. We selected weather records that covered the most populous regions, taking the mean where multiple weather stations were used. For Finland, data was extracted from the Kaisaniemi, Helsinki station [25]. For Mississippi, data was extracted from, Hattiesburg, Jackson, Memphis and Tupelo weather stations [26]. For Scotland, data was extracted for the West Scotland area [20]. We calculated annual anomalies and annual and seasonal means, where season 1 is December to February, season 2 March to May, season 3 June to August, and season 4 September to November.

The NTM infection, temperature and precipitation data were combined into a single dataframe. Temperature and precipitation lags were added, to represent these metrics for the preceding season or year against the NTM infection rates, based on lagged effects previously identified [8].

To represent future climate scenarios in the UK, we used the seasonal and annual predictions of precipitation and mean air temperature at 1.5 m in the 60 km resolution UK projection model from the Met Office Hadley Centre for 2024–2033 [27]. The data uses the 15 members of the Met Office Hadley Centre global 60km resolution model, HadGEM3-GC3.05 (PPE -15), under a low emissions scenario (RCP 2.6). It was processed similarly to the training and validation dataframe, for seamless integration, to create an additional test dataframe. This was done both on a regional resolution, using the 12 administrative regions provided by the Met Office Hadley Centre, and as a country as a whole.

We calculated Pearson’s correlation coefficient (R^2^) between the NTM incidence rates and seasonal precipitation and temperatures metric for Finland and Mississippi. The test was repeated for the seasonal and annual temperature and precipitation lags.

We used a Random Forest Regressor Model, previously found to have the highest predictive power for modelling nontuberculous mycobacterial pulmonary disease in Germany [28], consisting of 500 estimators and a maximum depth set at 3. The dataframes were split into training, validating and testing datasets for our model. The model was trained on a stratified subset of 50% of the data frame containing climate and NTM infections rates, from Finland, Mississippi and Scotland, in a supervised learning approach to recognise patterns. All spatial and temporal data (such as region and date) were removed to focus solely on associations between environmental conditions and NTM incidence rather than temporal trends. The model was validated and improved using a separate subset of 25% of the data, including feature permutation and parameter finetuning. The final model was tested on the remaining unseen 25% of the data. From this we were able to assess accuracy using three different metrics (mean relative error, mean squared error and mean absolute error), extract feature importance for each climate variable, and interrogate the resulting predictions.

The model with the highest accuracy was then applied to the UK forecast climate data dataset (both for the whole UK and the 12 regions) to forecast both predicted NTM incidence and the percentage increases expected over the next 10 years. As this dataset refers to future dates, there is no NTM data to assess the accuracy of these hypothetical predictions, but they can indicate relative and regional increases. Forecast results were appended to administrative region shapefiles associated with the UKCP data (Met Office Hadley Centre, 2023) and maps generated using QGIS.

## Results

**Fig 1:**
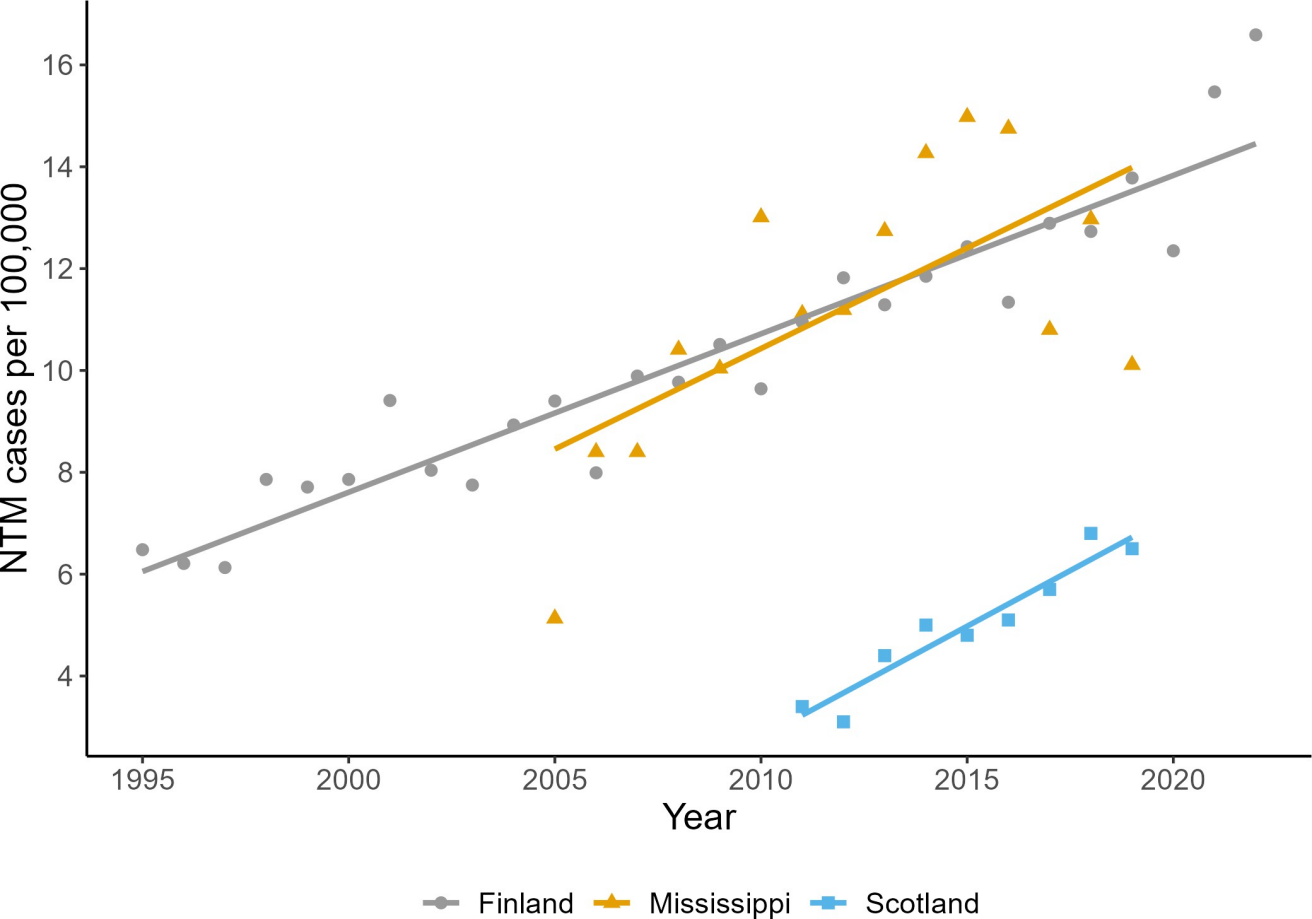
Recorded NTM infection rates are increasing for Finland (grey), Mississippi (orange) and Scotland (blue). The fitted lines (least squares, linear regression) show the trend in NTM infection rates. R2 = 0.9 (Finland), 0.43 (Mississippi), 0.91 (Scotland). (Data from Finnish Institute for Health and Welfare, Mississippi State Department of Health, Open Forum Infectious Diseases).

In regions where NTM infections are notifiable, there are significant increases in NTM infection rates (Fig 1). NTM infection rates have significantly (p<0.05) increased across all three regions providing data (Finland, Mississippi and Scotland), with R^2^ values of 0.96, 0.66 and 0.95 respectively. The trends of NTM infection rates show an average annual increase of 4.2% (Finland), 6.6% (Mississippi) and 9.5% (Scotland). In Finland, the total increase over 28 years was 112.6%, in Mississippi it was 99.79% over 16 years and in Scotland 75.65% over 8 years.

Analysis of weather records show the average temperatures and precipitation rates have steadily increased in these regions over the same time periods that NTM infection rates have increased (S2 Fig). Average temperature has increased annually for all regions, notably Finland and Mississippi. Average precipitation has increased annually for all regions, notably Mississippi and West Scotland. Interrogation of the UK climate projections found that projected climate change in the UK is likely to see similar increases in annual temperatures (particularly in terms of warmer summers) and higher intensity heavy rainfall events (also in summer), with regional variation [27].

**Fig 2:**
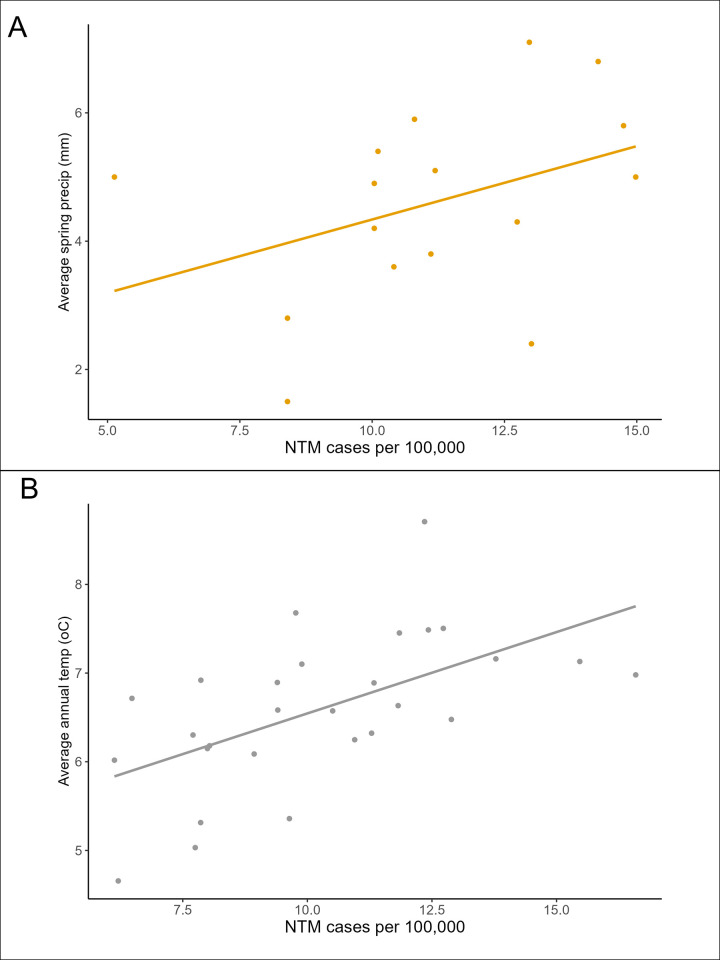
Ordinary least squares linear regression models with fitted regression line identifying environmental associations of NTM infection rates. Mississippi NTM infection rate data against average daily spring precipitation (A), and Finland NTM infection rate data against average annual temperature (B).

NTM infection rates are positively correlated with precipitation (in Mississippi) and temperature (in Finland). In Mississippi, NTM infection rates show strong positive correlation with daily precipitation (Fig 2A), particularly spring precipitation rates (R^2^ = 0.81). Summer precipitation in Mississippi is a strong predictor of NTM infections in summer and autumn seasons. In Finland, NTM infection rates show positive association with average annual temperatures (R^2^ = 0.51) (Fig 2B). The previous annual temperature and spring temperatures also positively correlate with NTM infection rates.

The Random Forest Classifier, when tested on the unseen 25% dataset (containing data points from Finland, Mississippi and Scotland), has a mean relative error of 0.19 per 100,000 people (with the lowest mean relative errors seen for the Scottish data at 0.11). The average absolute error is –1.01 per 100,000 people, slightly underestimating NTM incidence (S1 Table). Interrogation of each environmental metric used to train the model found that features, in order of importance, are: (i) average summer temperatures, (ii) the previous year temperature anomaly, and (iii) the spring daily precipitation (S3 Fig).

**Fig 3:**
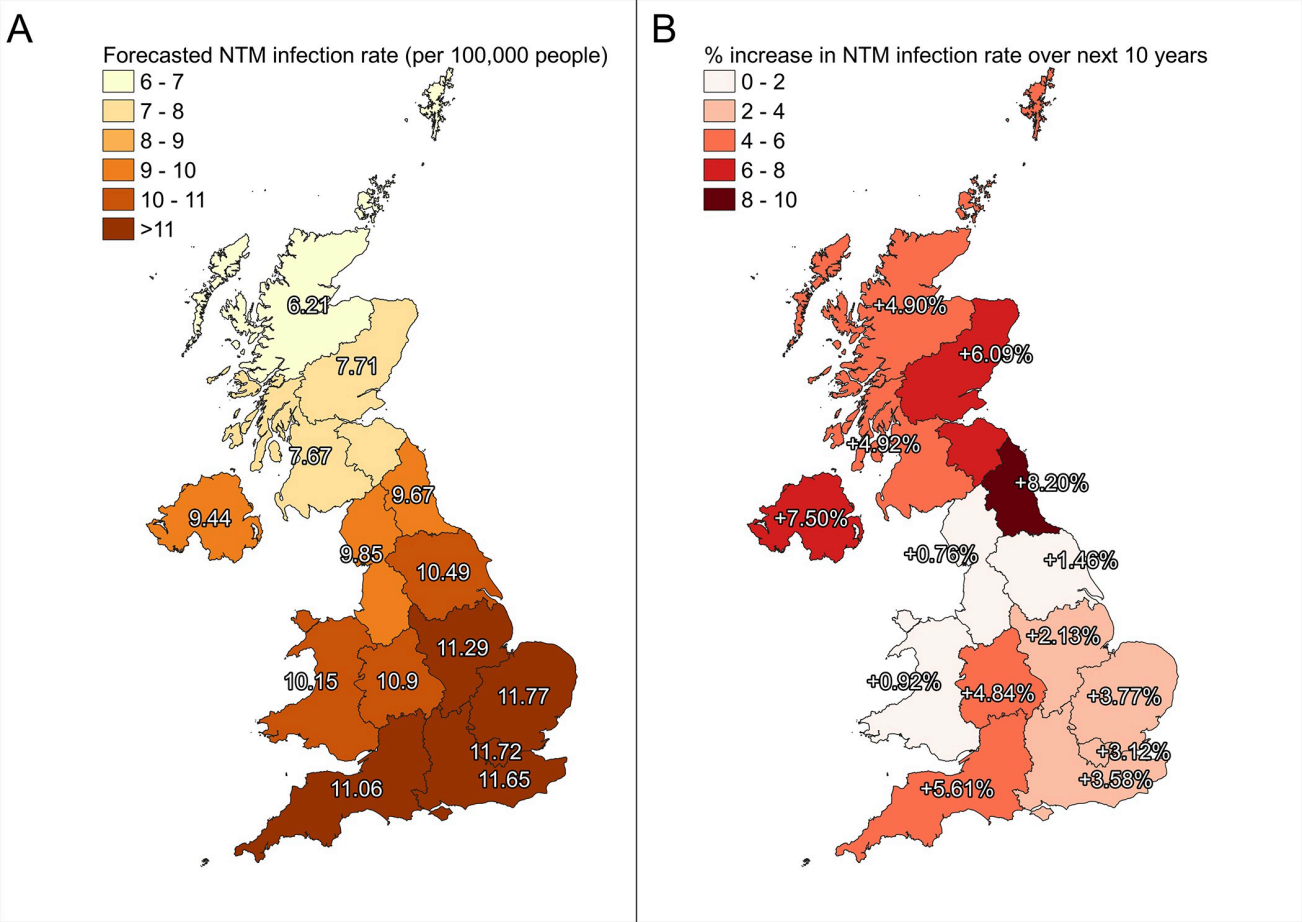
Projected regional model forecasts for NTM infections per 100,000 in the UK based on climate projections and a trained Random Forest Regressor. NTM infection rate per 100,000 people averaged over 2024–2033 (A), and the projected % increase over this time period (B). Shapefiles provided by Met Office Climate Projections [27] to complement regional projection areas.

Our model, based solely on UKCP projections, predicts a 6.2% increase in suitability for NTM across the UK from 2023 to 2033 and an increase in annual NTM infection rates to an average of 10.2 cases per 100,000 people across the UK (S2 Table). This is a 33.8% increase in NTM infections since last reported UK surveillance data in 2012. Regional application of our models show that southern regions will have the highest NTM infection rates (Fig 3). By 2033, NTM incidence in East England, London, and South-East England will have increased to 11.8, 11.7 and 11.7 cases per 100, 000 people respectively. The highest increases in NTM infection rates over the next 10 years will occur in North-East England, Northern Ireland, and East Scotland, at 8.2, 7.5, 6.1% respectively (Fig 3). We found average summer temperatures to have a strong positive climate association with predicted NTM infection rates (S4 Fig).

## Discussion

Our finding that NTM rates were positively associated with increased seasonal precipitation in Mississippi supports previous conclusions of correlated precipitation and NTM infection rates in Queensland, Australia [8]. In Mississippi, the precipitation rates in Spring, the rainiest season in the northern hemisphere, had the strongest correlation with NTM rates. In Mississippi specifically, high precipitation and upstream winter-spring transition snow-melt cause most severe flooding events in Spring [26]. Flooding associated with high precipitation likely facilitates transport of NTM [29, 30], contaminating drinking water sources [12].

Our finding that temperature and NTM infection rates correlate is expected, given that abundance of *Mycobacterium* species in natural [31] and municipal [13, 14] waters have been observed to increase with temperature. Laboratory tests show that NTM survive at temperatures as low as 15°C but thrive at temperatures over 28°C [16]. Warmer air temperatures can raise the temperature of water sources, particularly in pipes and water storage tanks, increasing suitability for NTM survival and therefore the abundance. This in turn increases the possibility of transmission to the host and the development of NTM infections. Mississippi’s average temperature range across the year (8 to 31°C) is warmer than Finland’s (-5 to 21°C in Helsinki) [32]. Given that Mississippi’s temperature often sits within the optimum suitability range for NTM, it is likely that variation in precipitation becomes a more important driver of NTM infection rates than temperature variation. NTM bacteria can be aerosolised both during periods of high temperatures, which increase water evaporation, and extreme weather events when soil and water mix, introducing new transmission routes by inhalation which may contribute to increases in NTM disease [33, 34].

The associations between NTM infection rates and environmental conditions are complex, and likely interact with other environmental conditions to have compounded effects. For example, NTM are found in fresh and brackish waters as they have a low tolerance of high salinity, limiting their survival in saltwater. High precipitation dilutes the salinity of the water bodies NTM inhabit, providing a survival advantage. Intense precipitation additionally increases surface run-off and transport of soil, facilitating the infiltration of water bodies and systems. This cascading effect can increase NTM abundance in water supplies. Further variables, such as salinity or soil moisture, could be included in future models to extract such complex, dynamic associations between water cycles and NTM in their environmental reservoirs.

The species-specific lag between exposure and clinical presentation adds further complexity. Slow-growing *Mycobacterium* species can take 8 to 10 months to display an incidence response after rainfall, whereas fast-growing species may only take 4 to 5 months [8]. We find that the species of *Mycobacterium* are not typically recorded during surveillance. This, combined with low overall incidence, currently hinders species-specific study. However, we find the relationships within our grouped-species data are sufficient to allow for forward modelling, with species-specific studies focusing on those of most concern becoming a long-term goal for future models.

Our model forecasts that the highest NTM infection rates in ten years will be in southern regions of the UK. This is likely due to regionally warmer summer temperatures and the positive associations of temperature with predicted NTM infection rates (S4 Fig). However, the greatest increases were seen in more northern regions. UK Climate Projections [27] predict summer average temperatures will exceed 15.5°C from 2030 in some northern regions (S5 Fig). These align with the same regions where NTM infection rates are predicted to increase most North-East England and Northern Ireland. This temperature represents the minimum temperature NTM need to survive and grow in water [16]. When average summer temperatures start to exceed this temperature threshold in these regions, NTM survival in the environment will increase leading to subsequent increases in infections. Future climate change is likely to continue to affect these environmental conditions, with increased global warm predicted with a high confidence in The Intergovernmental Panel on Climate Change (IPPC) 6th Assessment report [35]. The corresponding increased frequency and intensity of heat waves, extended warm seasons, and increases in precipitation in higher latitudes [36] will favour NTM growth.

Ultimately, the increases in NTM infections predicted by our model will have social and economic implications for the UK. The impact of the largest increases of NTM infections could be exacerbated by health inequalities found in these regions. These regions, notably North-East England, have poorer health outcomes compared to the rest of England [37]. Additionally, previous estimates suggest it costs around £9,727 per person per year to manage an NTM infection in the UK [38], with increased NTM infection rates carrying an economic burden. Future analysis should consider the inclusion of socio-economic or demographic data to enhance model predictions and provide assessments of specific populations at risk.

Other regions may offer more suitable comparisons for UK climate conditions, however NTM infection data is limited in such regions, restricting choices available for analysis. Similarly, UK NTM infection surveillance data is currently not collected and would be required to fully assess the accuracy of the modelled results, including refining the predictions and adjusting parameters accordingly. The model has an average relative error of 0.19 per 100,000 people when tested on known NTM infection rates, providing a crude uncertainty range for our predictions. Additionally, the percentage increases in risk provide informative estimates of increases in relative suitability across the time period. Such increases in suitability are pertinent for public health, preceding the implementation of surveillance systems.

The 75% increase in Scottish clinical NTM isolates over 8 years suggest other factors in addition to climate change could be contributing to the increase, such as host susceptibility or public health measures. These factors may contribute, but other studies suggest limited impact compared to the change in environmental factors that favour NTM growth and survivability.

1. Regarding increased susceptibility to infection with age as a contributor to the rise in NTM disease: a Korean study shows that from 2003 to 2016, NTM incidences increases in all age groups [39]. Additionally, some regions that have an aging population have not seen an observed increase in NTM infection, e.g. Denmark [40] and the Basque region (Spain) [41]. Together, these studies suggest the link between age and global NTM infection rates is uncertain.
2. Immunodeficiencies increase susceptibility to infectious disease, including PNTM (33). However, a study found that non-pulmonary NTM (non-PNTM) occurs across ages, ethnicities and gender, regardless of immunodeficiency status [42]. While the proportion of non-PNTM patients having taken immunosuppressants was considerably higher than in the general US population (3% [43]), the high proportion of non-PNTM cases not linked to immunosuppressant drug use indicates that it is not the solitary factor in non-PNTM. We found no comparable study for PNTM.
3. Alternatively, such high increases in clinical isolates such as in Scotland, could also be the result of increased clinical awareness or improving surveillance protocols.

Future, robust surveillance systems in the UK, and globally, would increase the predictive power necessary for forecasting trends of climate-associated infections.

## Conclusion

Our findings show associations between NTM infections incidence and increased precipitation and temperature. While other factors could contribute towards increases in NTM incidence, we argue these are not dominant drivers. The absence of compulsory NTM surveillance in the UK has previously limited prediction of incidence rate trends, however, our machine learning model, trained on international climate and surveillance data, provides a quantified projection of NTM incidence on a regional level across the UK based on climate change scenarios. The forecasted impacts of climate change in the UK will increase NTM infection rates. In the future, greater surveillance of NTM infections both in the UK and globally is highly recommended to facilitate further assessment of these impacts.

## Supporting information

S1 Fig Increases in NTM infections have been reported globally in the countries highlighted, but are only notifiable in a minority of countries. The following colours represent the status of NTM surveillance in these countries: green- areas where NTM is nationally notifiable; yellow- areas where NTM is regionally notifiable through local administrations; purple- areas where NTM is not notifiable but changes in NTM infections have been established from peer-reviewed literature. Created with mapchart.net.
(TIF)

S2 Fig Weather record trends in NTM notifiable regions for average monthly temperature (A) and precipitation (B).
(TIF)

S3 Fig Feature importance within random forest model. (TIF)

S4 Fig Predicted NTM incidence in the UK was positively correlated with average summer temperatures. Southern areas with the warmest summers were predicted the higher rates. (TIF)

S5 Fig Regional climate projections for average summer temperature. (TIF)

S1 Table Results of random forest model predictions of NTM infection rates on international unseen test dataset. (XLSX)

S2 Table Random forest model predictions for UK regions 2025–2033. (XLSX)
