# Supplementary figures and images for: Forecasting climate-associated non-tuberculous mycobacteria (NTM) infections in the UK using international surveillance data and machine learning
Source: PLOS Glob Public Health. 2024 Aug 19;4(8):e0003262. doi: 10.1371/journal.pgph.0003262 (PMC11332936; doi:10.1371/journal.pgph.0003262)

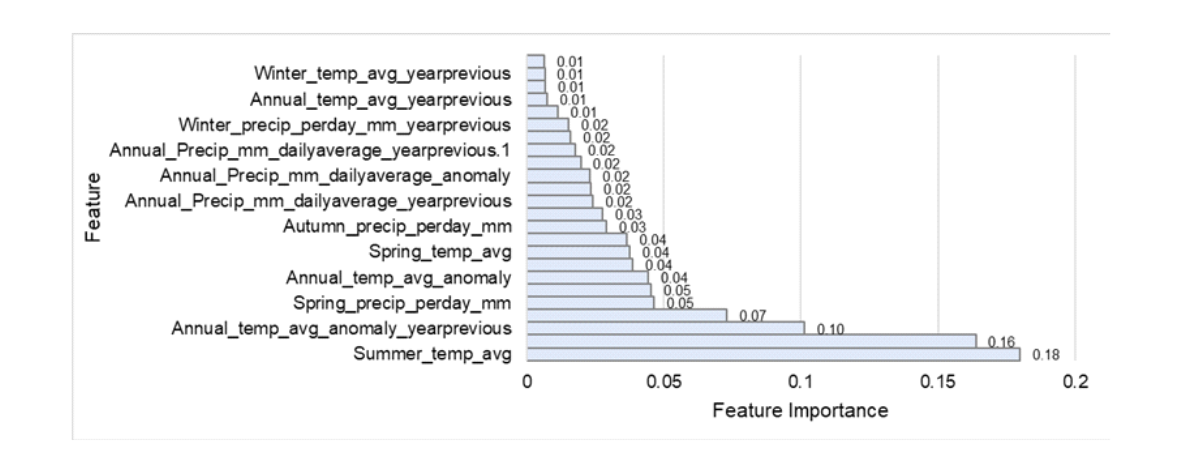

Supplement: S3 Fig — (TIF) [file pgph.0003262.s003.tif]

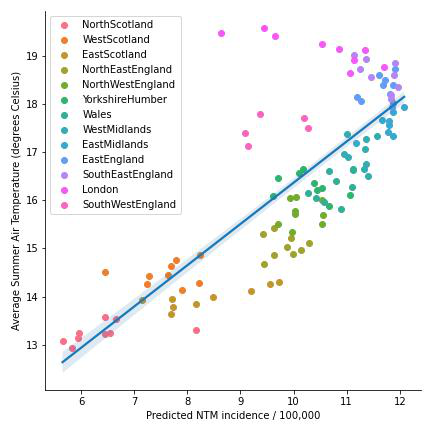

Supplement: S4 Fig — (TIF) [file pgph.0003262.s004.tif]

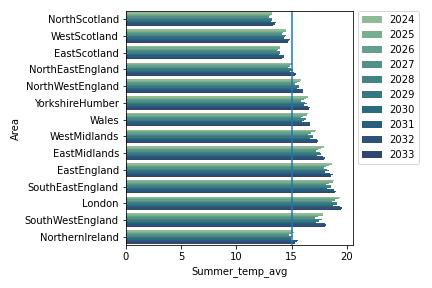

Supplement: S5 Fig — (TIF) [file pgph.0003262.s005.tif]
